# Supplementary material for: From crisis to recovery: Exploring the demand surge for mental health services in Alberta, Canada—A document-based policy analysis with an illustrative supply–demand simulation (2023–2024)
Source: PLOS Ment Health. 2026 Mar 25;3(3):e0000307. doi: 10.1371/journal.pmen.0000307 (PMC13016303; doi:10.1371/journal.pmen.0000307)
Supplement: S1 Text — (PDF) [file pmen.0000307.s001.pdf]

README for OSF Project: From Crisis to Recovery: Exploring the Demand Surge for Mental Health Services in Alberta, Canada

Project Overview

This OSF repository (DOI: 10.17605/OSF.IO/ESYFZ) supports the manuscript titled "From Crisis to Recovery: Mental Health Service Demand in Alberta, Canada — A Policy Analysis with Illustrative Supply–Demand Modeling (2023–2024)". The project evaluates Alberta's mental health system response to the COVID-19 pandemic using publicly available data from the Government of Alberta's 2023–2024 Mental Health and Addiction Annual Report and related literature. It includes empirical program metrics and an illustrative supply-demand simulation to examine directional shifts in service demand, capacity, and equilibrium under a zero-copayment design.

The analysis focuses on key interventions like CASA Mental Health classrooms, Virtual Opioid Dependency Program (VODP), recovery communities, and tele-mental health. Modeled outputs are synthetic and calibrated to trends (e.g., demand shift +27 services/month from Russell et al., 2024; capacity expansion ~30% derived from report). All materials are provided for transparency and reproducibility, consistent with CHEERS 2022 guidelines.

Registration Type: Open-ended (not a preregistration of empirical experiments). **License**: CC-BY Attribution 4.0 International. **Contributors**: Kola Adegoke (corresponding), Abimbola Adegoke, Deborah Dawodu, Ayoola Bayowa, Akorede Adekoya, Temitope Kayode, Mallika Singh, Olajide Alfred Durojaye, Abiodun Isola Aluko, Adeyinka Adegoke. **Created**: August 13, 2025. **Public Access**: Yes.

Files in This Repository

The following files are archived here to support the manuscript's findings. All data are from public sources; no proprietary or individual-level data are included.

- **Data_Assumptions_Alberta_Model_.xlsx**: Excel workbook with model inputs, parameters (e.g., base/low/high for demand +27/20/35, capacity 30%/20%/40%), sensitivity analyses, empirical extractions (Table 1 metrics), and dynamic formulas for reproducibility (e.g., $Q^* = \min(Q0 + \Delta D, Q0 * (1 + \Delta S))$).
- **Supply_Demand_Model_Tables_Figures.pdf**: Compiled tables (e.g., sensitivity Table 4) and figures (e.g., supply-demand curves, program reach bar chart) generated from the Excel workbook.
- **Graphical_Abstract_Alberta_Mental_Health_Crisis.pdf**: Visual summary of key interventions, empirical outputs, and modeled equilibrium shifts.
- **CHEERS_2022_guidelines.pdf**

How to Reproduce the Analysis

1. **Requirements**: Microsoft Excel (or compatible) for the workbook; no additional software needed.
2. **Steps**:
   - Download the Excel file.
   - Open "Parameter Table" sheet to view/adjust base values (e.g., demand increase calibrated from Russell et al., 2024).
   - Sheets like "Modeled Outputs" and "Sensitivity Analysis" auto-update with formulas (e.g., post-demand = pre + ΔD; Q^* computes capacity-constrained volume).
   - Recreate figures using built-in Excel charts or export data for tools like Matplotlib.
   - Empirical data in "Table1_ProgramMetrics" are direct extractions from the Annual Report (pages noted); verify via the report URL in "Data Sources" sheet.
3. **Notes on Modeling**: Simulations are illustrative (not predictive). Change parameters to test sensitivities; e.g., high demand + low capacity shows unmet need.

Data Sources and Citations

- Primary: Alberta Mental Health & Addiction Annual Report 2023–2024 (publicly available at linked URL).
- Secondary: Russell et al. (2024) for utilization trends; Folland et al. (2024) for economics framework.
- Full references in manuscript.

Contact

For questions, contact the corresponding author: Kola Adegoke (kola.adegoke01@utrgv.edu).

This repository ensures open access to materials for replication and policy discussion. Last updated: January 01, 2026.
