# Supplementary material for: From crisis to recovery: Exploring the demand surge for mental health services in Alberta, Canada—A document-based policy analysis with an illustrative supply–demand simulation (2023–2024)
Source: PLOS Ment Health. 2026 Mar 25;3(3):e0000307. doi: 10.1371/journal.pmen.0000307 (PMC13016303; doi:10.1371/journal.pmen.0000307)
Supplement: S1 Checklist — (DOCX) [file pmen.0000307.s003.docx]

**CHEERS 2022 Checklist**

Manuscript: From Crisis to Recovery: Exploring the Demand Surge for Mental Health Services in Alberta, Canada — A document-based policy analysis with an illustrative supply–demand simulation (2023–2024)

| **Section** | **Item** | **Guidance for reporting** | **Reported in section** |
| --- | --- | --- | --- |
| TITLE | 1 | Identify the study as an economic evaluation and specify the interventions being compared. | Title |
| ABSTRACT | 2 | Provide a structured summary that highlights context, key methods, results and alternative analyses. | Abstract |
| INTRODUCTION | 3 | Give the context for the study, the study question and its practical relevance for decision making in policy or practice. | Introduction |
| METHODS | 4 | Indicate whether a health economic analysis plan was developed and where available. | N/A (illustrative simulation) |
| METHODS | 5 | Describe characteristics of the study population (such as age range, demographics, socioeconomic, or clinical characteristics). | Method 4.3; Disc. 6.2 (equity) |
| METHODS | 6 | Provide relevant contextual information that may influence findings. | Intro; Sec. 4.2 |
| METHODS | 7 | Describe the interventions or strategies being compared and why chosen. | Intro 4.1 |
| METHODS | 8 | State the perspective(s) adopted by the study and why chosen. | Method 4.4 |
| METHODS | 9 | State the time horizon for the study and why appropriate. | Method 4.4 |
| METHODS | 10 | Report the discount rate(s) and reason chosen. | N/A (12-month horizon; Method 4.4) |
| METHODS | 11 | Describe what outcomes were used as the measure(s) of benefit(s) and harm(s). | Method 4.3 |
| METHODS | 12 | Describe how outcomes used to capture benefit(s) and harm(s) were measured. | Method 4.2; 4.3 |
| METHODS | 13 | Describe the population and methods used to measure and value outcomes. | N/A |
| METHODS | 14 | Describe how costs were valued. | N/A |
| METHODS | 15 | Report the dates of the estimated resource quantities and unit costs, plus the currency and year of conversion if relevant. | Method 4.4 (CAD, 2023–2024) |
| METHODS | 16 | If modelling is used, describe in detail and why used. Report if the model is publicly available and where it can be accessed. | Method 4.4; OSF workbook |
| METHODS | 17 | Describe any methods for analysing or statistically transforming data, any extrapolation methods, and approaches for validating any model used. | Method 4.3; 5.3 |
| METHODS | 18 | Describe any methods used for estimating how the results of the study vary for sub-groups. | N/A |
| METHODS | 19 | Describe how impacts are distributed across different individuals or adjustments made to reflect priority populations. | Sec. 6.2 |
| METHODS | 20 | Describe methods to characterize any sources of uncertainty in the analysis. | Sec. 5.3; Table 4 |
| METHODS | 21 | Describe any approaches to engage patients or service recipients, the general public, communities, or stakeholders in the design of the study. | N/A; 4.1 |
| RESULTS | 22 | Report all analytic inputs (e.g., values, ranges, references) including uncertainty or distributional assumptions. | Sec. 4.3; OSF |
| RESULTS | 23 | Report the mean values for the main categories of costs and outcomes of interest and summarise them in the most appropriate overall measure. | Sec. 5.1–5.2; Tables 1–3 |
| RESULTS | 24 | Describe how uncertainty about analytic judgments, inputs, or projections affects findings. Report the effect of choice of discount rate and time horizon, if applicable. | Sec. 5.3; Table 4 |
| RESULTS | 25 | Report on any difference patient/service recipient, general public, community, or stakeholder involvement made to the approach or findings. | N/A; 4.1 |
| DISCUSSION | 26 | Report key findings, limitations, ethical or equity considerations not captured, and how these could impact patients, policy, or practice. | Sec. 6.1–6.3; Limitation 8 |
| OTHER RELEVANT INFORMATION | 27 | Describe how the study was funded and any role of the funder in the identification, design, conduct, and reporting of the analysis. | No funding |
| OTHER RELEVANT INFORMATION | 28 | Report authors’ conflicts of interest according to journal or International Committee of Medical Journal Editors requirements. | Yes |

*Notes: Items marked “N/A” are not applicable because this study is a document-based policy analysis with an illustrative (non-CEA) supply–demand simulation, and does not estimate costs/QALYs/ICERs.*
