# Supplementary figures and images for: From crisis to recovery: Exploring the demand surge for mental health services in Alberta, Canada—A document-based policy analysis with an illustrative supply–demand simulation (2023–2024)
Source: PLOS Ment Health. 2026 Mar 25;3(3):e0000307. doi: 10.1371/journal.pmen.0000307 (PMC13016303; doi:10.1371/journal.pmen.0000307)

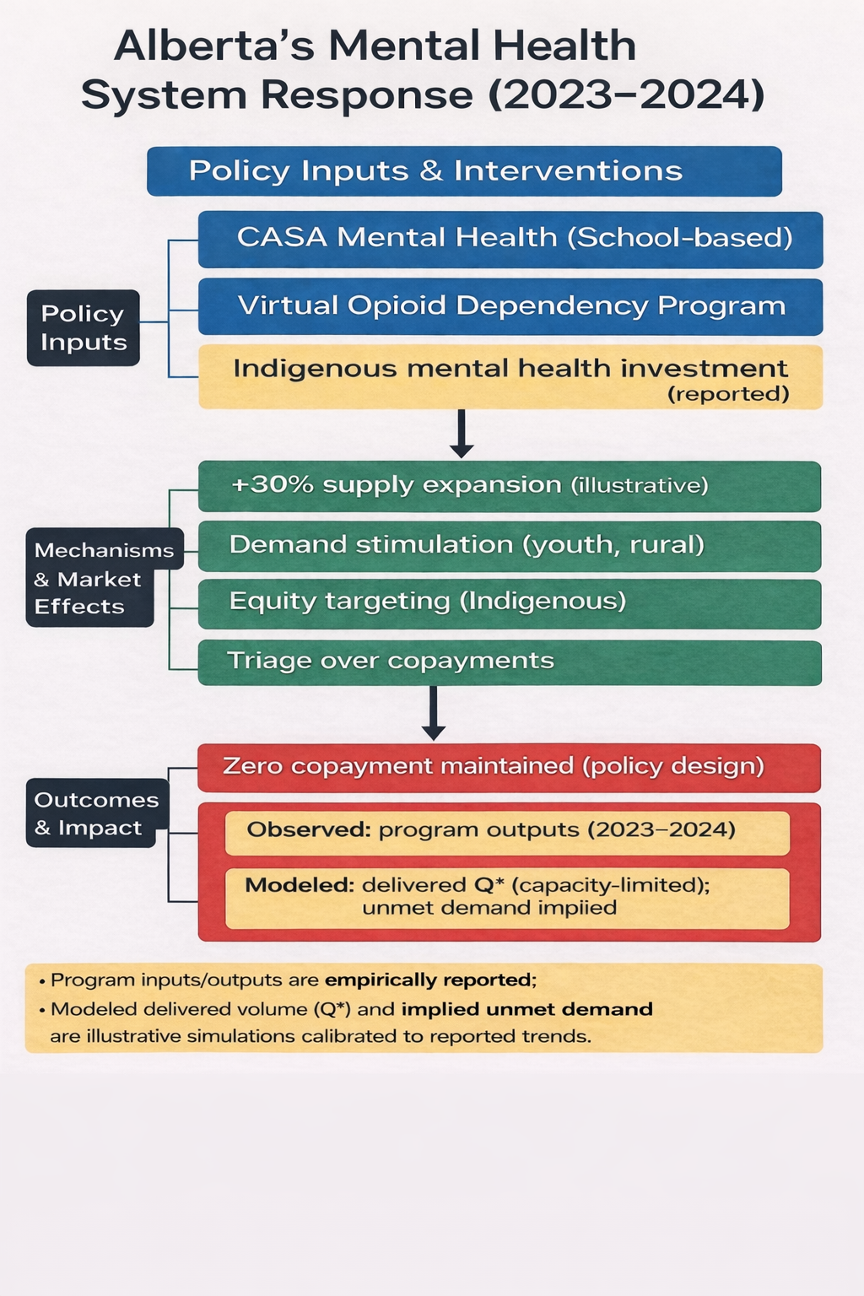

Supplement: S1 Fig — This graphical abstract summarizes key publicly funded interventions and empirically reported program delivery outputs from Alberta’s 2023–2024 reporting, alongside an illustrative supply–demand framework depicting the directional mechanics of equilibrium under a zero-copayment design. Modeled quantities shown in the equilibrium pathway are simulations calibrated to reported trends and should not be interpreted as observed prices or system-wide utilization. (TIF) [file pmen.0000307.s004.tif]
