# Supplementary material for: From crisis to recovery: Exploring the demand surge for mental health services in Alberta, Canada—A document-based policy analysis with an illustrative supply–demand simulation (2023–2024)
Source: PLOS Ment Health. 2026 Mar 25;3(3):e0000307. doi: 10.1371/journal.pmen.0000307 (PMC13016303; doi:10.1371/journal.pmen.0000307)
Supplement: Attachment — Submitted filename: Rebuttal_Letter_Plos_Mental_Health 2.pdf [file pmen.0000307.s006.pdf]

KOLA ADEGOKE, DrPH (Cand.), PMP®, CPHIMS™, FACHE©
kolaadegoke17@gmail.com | 587-936-7155
www.linkedin.com/in/kola-adegoke | ORCID: 0009-0005-5615-1152

28th July 2025

Response to Reviewers

Manuscript ID: PMEN-D-25-00038
Title: *From Crisis to Recovery: Exploring the Demand Surge for Mental Health Services in Alberta, Canada*
Corresponding Author: Dr. Kola Adegoke

To: The Editors, PLOS Mental Health

Attention: Dr. Olumide Thomas Adeleke

Dear Dr. Adeleke and Reviewers,

We sincerely thank you for your thoughtful feedback and the opportunity to revise our manuscript. We have carefully addressed all reviewer and editorial comments in detail and made substantial improvements to the manuscript.

Importantly, this revised manuscript represents a substantially enhanced version of the original submission, with additional contributors whose expertise significantly strengthened the modeling, policy analysis, and empirical validation. As such, **three additional co-authors** have been added who actively participated in the revision process.

All listed authors meet the ICMJE authorship criteria and have made meaningful contributions to the conception, drafting, or revision of this manuscript.

Editorial Requirements:

Issue	Response
1. Financial Disclosure	Updated: "The authors received no specific funding for this work."
2. Competing Interests	Verified and confirmed as accurate.
3. Data Availability	Revised to: "All data are in the manuscript and/or supporting information files."
4. Manuscript Format	Submitted as a .docx file with tracked and clean versions.
5. Figure File Format	All figures are provided separately in .tif format per journal requirements.
6. Figure 1 Upload	Uploaded and cited correctly in the revised manuscript.
7. Reviewer-Recommended Citations	Evaluated and integrated where relevant.

Reviewer #1 Comments and Our Responses

1. **Missing Citation – Alberta's 2023–24 Annual Report**
   Added citation [3] supporting both investment and VODP data points.
2. **VODP Usage – Missing citation**
   Same citation [3] applies.
3. **Typo – "linking" → "linkage"**
   Corrected as advised.
4. **Stable Equilibrium – Citation missing**
   Now cited as: Folland et al. (2024) [4].
5. **Youth Wait Times – Citation needed**
   Supported using Talarico (2024) [1] and Russell et al. (2024) [2].
6. **Results and Discussion – Suggest separation**
   Separated into: Section 5 (Results) and Section 6 (Discussion).
7. **Provincial Comparison Suggested**
   A comparison with Ontario's Centre of Excellence is included in Section 6.3, with citation [7].

Reviewer #2 Comments and Our Responses

Strengths:

We gratefully acknowledge the reviewer's commendation of our study's relevance, framework, use of real-world data, and visual clarity.

Methodological Clarifications:

1. **Data Transparency – Tables 1 & 2**
   Captions now state: "modeled using extrapolated data from sources [3,4]." Explained in Section 4.1.
2. **Assumptions Made – Need Clarity**
   Assumptions listed in Section 4.1, including:
   - Uniform provider quality
   - Proportional capacity expansion
   - Flat pricing schemes
3. **Counterfactual Discussion Needed**
   ✔ *Section 6.2 expanded to reflect plausible outcomes without intervention (referencing WHO [17]).*

Theoretical Enhancements:

1. **Inclusion of Health Economics Concepts**
   Section 3.3 now integrates:
   - Moral hazard
   - Adverse selection
   - Information asymmetry
   - Provider-induced demand (expanded discussion)

2. **Equity Considerations for Rural & Indigenous Populations**
   Expanded in Section 3.4.2 and Section 7. Focused examples and implications included.

Empirical Evidence:

1. **Clinical Outcomes**
   Section 6.1 addresses outcome limitations and references Talarico [1] for observed impacts.
2. **Cost-Effectiveness Analysis**
   Section 6.3 now includes fiscal sustainability projections and insights into marginal returns.
3. **Long-Term Sustainability**
   Explored in Section 7 with references to public-private partnerships and multi-year budgeting.

Structural Improvements:

1. **Literature Review Expansion**
   Now includes broader comparisons with Canadian (Ontario), Australian, UK, and WHO data.
2. **Abstract – Redundancy Removed**
   Rewritten for clarity and conciseness.

Visual Enhancements

Figures 1–5 revised with consistent formatting.
All captions now use:
Source: Modeled using Alberta Health [3], Russell et al. [2], and Folland et al. [4].

Closing Statement

We appreciate the opportunity to revise this work and are grateful for the detailed and constructive comments you provided. We believe the manuscript is now significantly stronger and better aligned with PLOS Mental Health's standards and scope.

We hope you will find the revised version suitable for publication.

Sincerely,

Dr. Kola Adegoke
On behalf of all authors
Email: kadegoke@student.touro.edu
