# Supplementary material for: From crisis to recovery: Exploring the demand surge for mental health services in Alberta, Canada—A document-based policy analysis with an illustrative supply–demand simulation (2023–2024)
Source: PLOS Ment Health. 2026 Mar 25;3(3):e0000307. doi: 10.1371/journal.pmen.0000307 (PMC13016303; doi:10.1371/journal.pmen.0000307)
Supplement: Attachment — Submitted filename: Response to the Academic Editor and Reviewers.pdf [file pmen.0000307.s007.pdf]

Response to the Academic Editor and Reviewers

Manuscript title: *From Crisis to Recovery: Exploring the Demand Surge for Mental Health Services in Alberta, Canada — A document-based policy analysis with an illustrative supply–demand simulation (2023–2024)*

Manuscript ID: PMEN-D-25-00038R1

Corresponding author: Kola Adegoke

Overview of revisions

We thank the Academic Editor and reviewers for their constructive feedback. In the revised manuscript, we made major structural and reporting changes to improve transparency, internal consistency, and reproducibility. Key revisions include:

1. Reframed the work explicitly as a **document-based policy analysis** with an **illustrative supply–demand simulation** (not a cost-effectiveness evaluation).
2. Reorganized the **Results** to clearly separate **empirical (observed) program outputs** from **modeled (simulated) outputs**, with explicit labeling throughout.
3. Updated model calibration and interpretation to reflect a **capacity-constrained delivered volume framework** (base delivered **Q*** = 78 services/month), clarifying that when modeled demand exceeds deliverable capacity, the residual is interpreted as **unmet demand/queueing** rather than additional delivered utilization.
4. Added **deterministic one-way sensitivity analysis** and **multi-way scenario checks** to clarify uncertainty and robustness.
5. Updated **Data Availability** with an OSF DOI and provided replication materials (workbook(s), parameter table with bounds, figures, and a short README).: **Registration DOI 10.17605/OSF.IO/6DBFU**, Associated project **https://osf.io/e6n7w**
6. Moved limitations out of Methods into a dedicated **Limitations** section and reduced reliance on bullet points by strengthening narrative explanation.

Editor Comments

Editor Comment 1: Move "Limitations" out of Methods

Response: Done. We removed limitations content from Methods and relocated it to a dedicated Limitations section following the Discussion/Policy Implications.

Change made: Removed prior "Limitations" content from Methods Section 4; added **Section 8. Limitations**.

Editor Comment 2: Reduce overuse of bullet points

Response: Done. We converted most bullet lists to narrative paragraphs and retained structured numbering only where it improves readability (e.g., clearly labeled assumptions).

Change made: Sections 4.3, 7, and 9 were rewritten to narrative form with selective numbering.

Editor Comment 3: Results combine observed findings with modeled outputs; separate clearly

Response: Done. The Results section now explicitly distinguishes:

- **5.1 Empirical results** (directly observed program delivery metrics)
- **5.2 Modeled results** (illustrative supply–demand simulation outputs)
- **5.3 Uncertainty/robustness** (deterministic sensitivity + scenario checks)

  We added explicit statements that model values are simulations, **not observed prices or system-wide utilization counts**.

  Change made: Section 5 reorganized; tables and figures retitled/re-captioned to indicate empirical vs modeled outputs.

Reviewer #3

R3.1 Data & Code Availability (Required): Deposit data and analysis code; update Data Availability Statement with DOI; add README

Response: Completed. We created an OSF repository containing extracted summary inputs, analysis workbook(s), figure files, parameter tables with bounds, and a short README with step-by-step reproduction instructions. The Data Availability Statement includes the OSF DOI.

Change made: Data Availability Statement updated; OSF DOI included: **Registration DOI 10.17605/OSF.IO/6DBFU**, Associated project **https://osf.io/e6n7w**

R3.2 Methods reporting perspective, time horizon, currency/price year, inflation/PPP, software/version, parameter table with sources, discounting statement

Response: Completed. Methods now state an analytic perspective (provincial public payer/system), time horizon (12 months), currency (CAD), reporting period (2023–2024), and that no inflation/PPP adjustments were applied. Discounting is stated as not applicable (12-month horizon). Software/version and parameter table sourcing are specified.

Change made: Methods Sections 4.3–4.4 updated; parameter table referenced in Supporting Information/OSF.

R3.3 Uncertainty & robustness: add deterministic sensitivity analysis; consider scenario analysis

Response: Completed. We added deterministic one-way sensitivity analyses over key parameters (baseline utilization, demand increase magnitude, capacity expansion magnitude) and included multi-way scenario checks.

Change made: Results Section 5.3; added **Table 4** (sensitivity + scenario checks).

R3.4 Results vs Discussion: separate quantitative outputs from interpretation/limitations

Response: Completed. Quantitative outputs are presented in Results; interpretation, policy implications, and limitations are presented in subsequent sections.

Change made: Sections 6–10 revised accordingly.

R3.5 Figures & Tables: ensure figures submitted separately; tables include units/denominators/price year

Response: Completed. Figures are prepared as separate submission files. Tables clarify units/definitions and distinguish empirical vs modeled outputs. The unit-cost proxy is labeled as a modeling convenience, not an observed price.

Change made: Table titles/notes and figure captions revised for transparency.

R3.6 Reporting guidelines: submit the CHEERS 2022 checklist

Response: Completed/Prepared. We completed the CHEERS 2022 checklist and mapped each item to manuscript sections, which are included as Supporting Information.

Change made: CHEERS checklist added as a supplement.

R3.7 Ethics statement: clarify publicly available non-identifiable data; no ethics approval/consent required

Response: Completed. We clarified that only publicly available, aggregate, non-identifiable data were used, with no access to individual-level data or contact with participants.

Change made: Ethics/Consent statement updated in the front matter.

R3.8 AI/LLM transparency statement

Response: No AI tools were used for writing or analysis. All content was developed, checked, and verified by the authors.

R3.9 References: use Vancouver formatting; include DOIs where available

Response: Completed. References were reformatted in Vancouver style, and DOIs added where available.

Change made: Reference list and in-text citations updated.

Reviewer #4

R4.1 Abstract conclusion more actionable

Response: Completed. We revised the Abstract Conclusion to emphasize policy implications and sustainability requirements.

Change made: Abstract Conclusion revised.

R4.2 Introduction link to global mental health policy trends

Response: Completed. We strengthened the Introduction to situate Alberta's response within post-pandemic trends (tele-mental health, community-based recovery, equity).

Change made: Introduction revised.

R4.3 Clarify policy frameworks/economic theories underpinning analysis

Response: Completed. We clarified how the supply–demand framework functions as an illustrative systems lens under a zero-copayment context and defined key terms.

Change made: Sections 3.1 and 4.4 revised.

R4.4 Address external factors (population growth/economic shifts) and why excluded

Response: Added. We note that the analysis is constrained to publicly reported outputs and uses a directional illustrative simulation rather than incorporating macro-level drivers; this is discussed as a limitation.

Change made: Assumptions and Limitations updated.

R4.5 Geographic variability and generalizability

Response: Expanded. We added discussion of urban–rural differences and identified lack of stratified outcome data as a limitation.

Change made: Discussion and Limitations revised.

R4.6 Expand discussion of PPPs

Response: Revised. We present partnerships as conditional and include governance safeguards (quality oversight, accountability, equity measurement), avoiding any implication that PPPs are inherently

beneficial.

Change made: Policy Implications revised.

R4.7 Limitations of review process / publication bias

Response: Clarified. We explicitly note that reliance on public reporting may introduce reporting/availability bias and incomplete capture of implementation challenges.

Change made: Limitations revised.

R4.8 More specific recommendations for future research

Response: Expanded. We added program-specific priorities (CASA, VODP, recovery communities, tele-mental health), emphasizing outcomes beyond service volume and equity audits.

Change made: Future Research revised.

Reviewer #5

We thank Reviewer #5 for their detailed feedback. We addressed the reviewer's core concerns by:

1. Clarifying the research question and analytic framing (policy analysis + illustrative model boundaries).
2. Improving definitions and Alberta program context earlier in the manuscript.
3. Strengthening the separation between empirical outputs and modeled results, including the capacity-constrained interpretation of delivered volume.

   A detailed point-by-point response to Reviewer #5 is provided in the attached document.
